# G-Quadruplexes at Telomeres: Friend or Foe?

**DOI:** 10.3390/molecules25163686

**Published:** 2020-08-13

**Authors:** Tracy M. Bryan

**Affiliations:** Children’s Medical Research Institute, Faculty of Medicine and Health, University of Sydney, Westmead, NSW 2145, Australia; tbryan@cmri.org.au

**Keywords:** G-quadruplex, telomere, telomerase

## Abstract

Telomeres are DNA-protein complexes that cap and protect the ends of linear chromosomes. In almost all species, telomeric DNA has a G/C strand bias, and the short tandem repeats of the G-rich strand have the capacity to form into secondary structures in vitro, such as four-stranded G-quadruplexes. This has long prompted speculation that G-quadruplexes play a positive role in telomere biology, resulting in selection for G-rich tandem telomere repeats during evolution. There is some evidence that G-quadruplexes at telomeres may play a protective capping role, at least in yeast, and that they may positively affect telomere maintenance by either the enzyme telomerase or by recombination-based mechanisms. On the other hand, G-quadruplex formation in telomeric DNA, as elsewhere in the genome, can form an impediment to DNA replication and a source of genome instability. This review summarizes recent evidence for the in vivo existence of G-quadruplexes at telomeres, with a focus on human telomeres, and highlights some of the many unanswered questions regarding the location, form, and functions of these structures.

## 1. Introduction

Telomeres are the DNA-protein complexes that cap the ends of linear eukaryotic chromosomes. In many species, telomeric DNA consists of tandem copies of a short guanine-rich repeat, containing a run of two to four consecutive guanines [1]. The telomere repeat sequence in vertebrates, trypanosomes, some fungi and some slime molds is TTAGGG. Many ciliated protozoa have repeats containing four guanines, while budding yeast tend to have longer and more irregular repeats [1]. One of the primary functions of telomeres, first recognized in the 1930s by Hermann Muller and Barbara McClintock in experiments with *Drosophila* and maize, respectively [2,3], is to distinguish natural chromosome ends from broken chromosomes, and thus protect the ends from DNA repair mechanisms leading to repair, recombination and fusion. Telomeres also serve as a gene-free buffer against the “end replication problem”, i.e., the inability of DNA polymerases to copy the very ends of chromosomes [4,5,6]. The latter property results in shortening of telomeres over time in human somatic cells [7]; unicellular organisms, germ cells, stem cells and most cancer cells have mechanisms to counteract this shortening, usually using the ribonucleoprotein enzyme telomerase [8,9,10,11].

Telomeres comprise a double-stranded region, of several kilobases (kb) in length in humans, terminating in a single-stranded overhang of the G-rich sequence. The discovery of the high conservation of G-rich sequences at telomeres suggested that the guanines may participate in secondary structures [12,13,14], and the first such structures were identified using single-stranded oligonucleotides representing the telomere sequences of ciliated protozoa [15,16,17]. It was found that, like other G-rich sequences [18], these telomeric sequences form into G-quadruplex (or G4) structures, in which four guanines form a planar array stabilized by Hoogsteen base-pairing (a G-quartet) [19], and multiple G-quartets stack on each other to form a stable, compact structure [16,17]. The ability of telomeric sequences to form into G-quadruplexes in vitro is conserved in highly divergent organisms, including other unicellular eukaryotes such as *Plasmodium* [20] and *Giardia* [21], humans and other organisms with the TTAGGG repeat [22,23], plants such as *Arabidopsis* [24,25], the budding yeast *Saccharomyces cerevisiae* [24,26] and invertebrates including the silkworm *Bombyx mori* [27]. Indeed, a systematic analysis of telomeric sequences from 15 divergent species showed that almost all of them have the capacity to form G-quadruplexes in vitro [24]. The only exceptions were two-guanine repeats from the yeasts *Schizosaccharomyces pombe* and *Candida guillermondii*, but these organisms have irregular telomere repeat sequences, and other *S. pombe* repeat permutations containing 3–4 guanines do form G-quadruplexes [28].

It is now more than 30 years since telomeric sequences were shown to form into secondary structures in vitro, yet many questions regarding the biological implications of this observation remain. This short review will highlight some of the many outstanding questions and areas for further research, particularly relating to the existence and functions of telomeric G-quadruplexes in human cells.

## 2. Direct Evidence for the Formation of G-Quadruplexes at Telomeres

**Figure 1:**
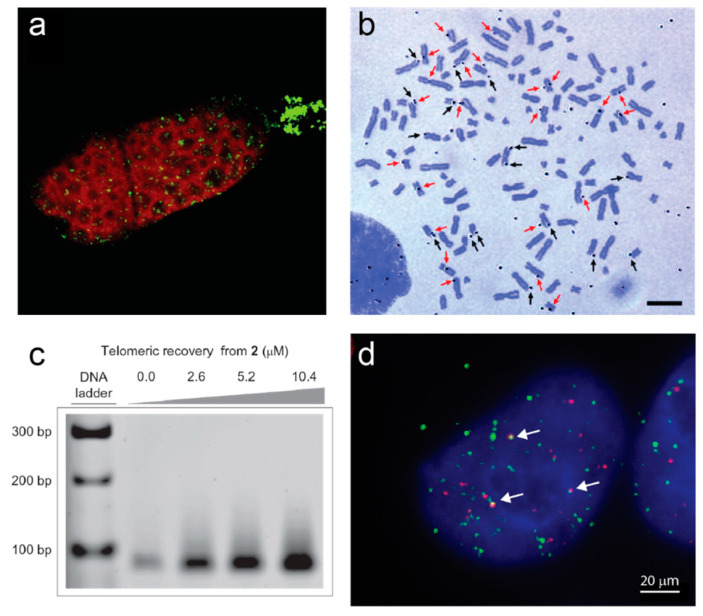
Examples of direct evidence for formation of G-quadruplexes at telomeres. (**a**) Immunofluorescence of a *Stylonychia lemnae* cell using an antibody raised against telomeric G-quadruplexes (green). DNA is counterstained in red; the replication band is the unstained region extending across the cell. Image from [35]. (**b**) Autoradiograph of metaphase spread of human T98G cells cultured with labeled G4 ligand ^3^H-360A for 48 h. Black arrows indicate silver grains on the terminal regions and red arrows indicate silver grains on the interstitial regions. Bar = 10 µm. Image from [36]. (**c**) Pull-down of telomeric DNA from human HT1080 cells using the indicated concentrations of a derivative of G4 ligand pyridostatin attached to an affinity tag (2). Genomic DNA was sheared into 100–300 bp pieces prior to pulldown, and telomeric sequences detected by PCR amplification. Reprinted by permission from Springer Nature [37]. (**d**) Immunofluorescence of a human 293T cell using the BG4 antibody against G-quadruplexes (green) together with fluorescence in situ hybridization against telomeric DNA (red). Arrows indicate G4-telomere colocalizations. Image by A.L. Moye and T.M. Bryan.

The first direct evidence for the formation of G-quadruplexes at telomeres in vivo came from studies of ciliated protozoa. These unicellular eukaryotes are distinguished by their unique nuclear morphology; they have two nuclei, a somatic “macronucleus” and a germline “micronucleus”. In a subset of ciliates known as hypotrichous ciliates, the genome of the macronucleus is amplified and fragmented into ~10^8^ gene-sized pieces, each of which carries a telomere at each end; they are therefore excellent model systems for the study of telomeres [29,30]. A single chain antibody generated in vitro against a G-quadruplex formed from the telomeric sequence of *Stylonychia lemnae* (TTTTGGGG) reacted specifically with the macronucleus of this ciliate [31]. A region of the macronucleus known as the replication band, where DNA replication and telomere elongation take place, was not recognized by the antibody, providing evidence that the G-quadruplexes detected by this antibody are resolved at the time of DNA replication and telomere extension, possibly to allow access to telomerase (Figure 1a). It had previously been demonstrated that the β subunit of the heterodimeric telomere-binding protein TEBP from the related ciliate *Oxytricha* is able to stimulate intermolecular G-quadruplex formation in vitro [32]; consistent with this, depletion of the β subunit from *Stylonychia* cells eliminated the immunofluorescence signal from the G-quadruplex antibody in vivo [33]. In S phase, the TEBP β subunit becomes phosphorylated, causing it to recruit telomerase together with a G-quadruplex-unwinding helicase to the telomeres, resulting in resolution of the telomeric G-quadruplexes [34,35]. This remains the most complete description of in vivo telomeric G-quadruplex dynamics.

In human cells, G-quadruplex-binding ligands provided the first visual demonstrations of G-quadruplexes at telomeres. A radiolabeled version of G4 ligand 360A was detected by autoradiography at the ends of human metaphase chromosomes (Figure 1b) [36], and the G4 ligand pyridostatin was conjugated to a biotin affinity tag to enable pulldown of telomeric G-quadruplexes from human genomic DNA (Figure 1c) [37]. A single-chain antibody with high specificity for G-quadruplexes (BG4) has enabled direct visualization of these structures across the genome of human cells; about 20−25% of the foci localize to telomeres (Figure 1d) [38]. Most of the BG4 nuclear foci are sensitive to DNase treatment; they increase in number during S-phase and decrease upon inhibition of DNA polymerase, providing evidence for an increased propensity for G-quadruplex formation during DNA replication [38]. Telomeric BG4 foci increase upon treatment of cells with G4-stabilizing ligands, including the parallel-specific G4 ligand NMM, implying that telomeric DNA has the capacity to form parallel G-quadruplexes in human cells [39]. The BG4 antibody has also enabled demonstration of the existence of G-quadruplexes at telomeres in the yeast *S. cerevisiae* [26]. Chromatin immunoprecipitation (ChIP) with BG4 followed by quantitative PCR of telomeric DNA showed that G-quadruplexes form at telomeres in this species; they were also enriched during late S phase [26].

Indirect support for the existence of G-quadruplexes at human telomeres has come from the many studies demonstrating the telomeric effects of the treatment of human cells with G-quadruplex stabilizing ligands (reviewed in [40,41]). These effects include the depletion of telomeric proteins TRF2 and/or POT1 from telomeres [42,43,44,45,46], degradation of the telomeric G-rich overhang [42,47,48], an increase in DNA damage signals at telomeres [44,45,46], and impaired replication of telomeres [49,50]. A ligand with specificity for parallel G-quadruplexes induced telomeric DNA damage foci, again suggesting that parallel G-quadruplexes can form at telomeres [51]. It should be borne in mind, however, that some of these effects may be indirect, caused by binding of the ligands elsewhere in the genome. For example, the promoter of the gene encoding the catalytic subunit of telomerase, hTERT, harbors G-quadruplexes [52], and G4-stabilizing ligands have been shown to decrease levels of hTERT expression [53,54,55], which may in turn lead to telomere deprotection [56,57]. G4-stabilizing ligands have also been shown to have widespread effects on gene expression, including genes involved in DNA damage pathways [58,59], likely due either to the enrichment of G-quadruplexes in gene regulatory regions [60,61], in response to the genome-wide DNA damage response triggered by G4 ligands [62,63], or as a result of epigenetic changes affecting gene expression [64,65]. Therefore, development of direct tools to detect G-quadruplex formation in cells, such as the antibody described above, is proving invaluable.

## 3. G-Quadruplexes in the Telomeric Single-Stranded Overhang

Telomeres terminate in a 3′ overhang of the G-rich sequence in all species for which this has been examined [66,67,68,69,70]. Ciliated protozoa have relatively short and well-defined telomeric overhangs of 14–21 nucleotides (nt) [68,71], which precludes their ability to form intramolecular G-quadruplexes involving four tracts of Gs; the structures detected with G4-specific antibodies are presumed to be dimeric or tetrameric, mediating interactions between telomeres [12,31,72]. Human telomeres, on the other hand, possess single-stranded overhangs of 20–400 nt [73,74], which is sufficient for formation of multiple intramolecular G-quadruplexes. However, there is no direct evidence as yet that G-quadruplexes form at telomeric overhangs in vivo, since the immunofluorescence techniques used to demonstrate their presence do not have sufficient resolution to discriminate between overhangs and the rest of the telomere.

**Figure 2:**
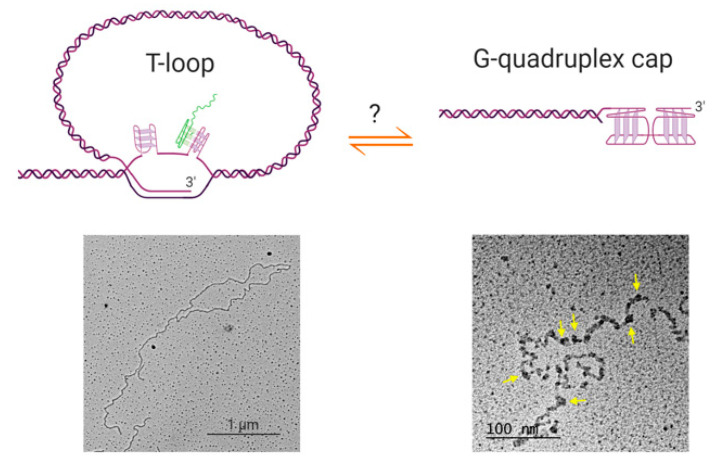
Possible relationships between t-loops and G-quadruplex structures. Top left: schematic of a t-loop formed by intercalation of a telomeric 3′ overhang into the duplex portion of a telomere; it is also possible for the other strand to participate in stabilizing the junction [81]. It is possible that G-quadruplexes could form in the displaced G-strand (the “D-loop”), or involving the 3′ overhang at the junction (not shown). It has been shown that RNA transcribed from telomeres (TERRA; green) localizes to the t-loop junction, possibly through DNA-RNA G-quadruplex formation [81]. Bottom left: electron microscopy image of a t-loop in genomic DNA isolated from human HeLa cells; image by Jack D. Griffith. Top right: it is possible that G-quadruplexes form at the telomeric overhang at times in the cell cycle when t-loops are resolved, although there is no direct evidence for this at present. Bottom right: Electron micrograph showing G-quadruplex formation in a long single-stranded telomeric fragment; image by Jack D. Griffith. Arrows indicate bead-like structures that represent higher-order interactions between multiple G-quadruplexes [82]. Figure created with BioRender.com.

Furthermore, if G-quadruplexes do form at human telomeric overhangs, they would need to compete with the other known higher-order telomeric structure, the t-loop [75]. T-loops are lariat structures in which the 3′ overhang invades an upstream double-stranded region of the telomere, forming a loop of several kilobases of DNA (Figure 2). These structures are widely conserved throughout evolution, having been found in mammalian and avian cells, the micronuclei of hypotrichous ciliates, the protozoan *Trypanosoma brucei*, the nematode *Caenorhabditis elegans* and plants [76,77,78]. While the ex vivo electron microscopy and super-resolution microscopy used to visualize t-loops do not directly report on numbers of t-loops in vivo, it has been estimated that a majority of telomeres in human cells form t-loops at any time [79,80]. The circumstances under which telomeric overhangs are free to form G-quadruplexes therefore remain to be determined. T-loops would need to be resolved during S-phase for telomere replication to occur; it is possible that during this part of the cell cycle, G-quadruplexes represent an alternative telomere-capping solution to the t-loop, preventing recognition of the telomere by the DNA damage machinery.

**Figure 3:**
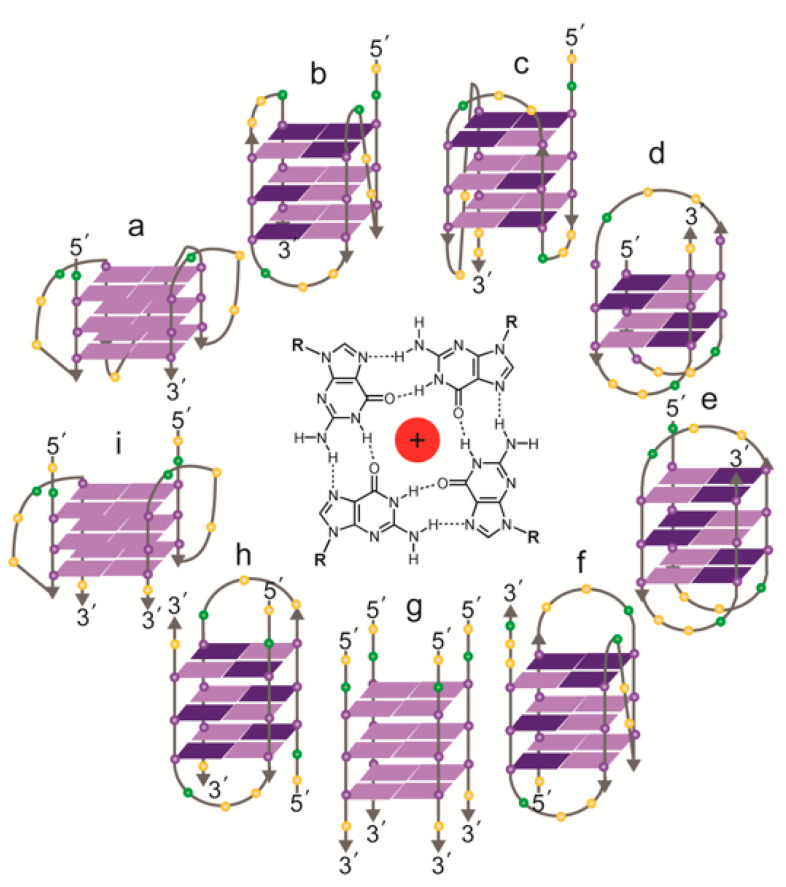
Topologies of solved structures of human telomeric G-quadruplexes, either intramolecular (**a**–**f**) or intermolecular (**g**–**i**). Centre: a G-quartet, comprising four guanines, stabilized by a central cation. (**a**) Crystal structure of AG_3_(T_2_AG_3_)_3_ in K^+^ (*parallel monomer*) [84]; (**b**) NMR structure of TAG_3_(T_2_AG_3_)_3_ in K^+^ (*hybrid form 1*) [85]; (**c**) NMR structure of TAG_3_(T_2_AG_3_)_3_TT or TTAG_3_(T_2_AG_3_)_3_TT in K^+^ (*hybrid form 2*) [85,87]; (**d**) NMR structure of G_3_T_2_A(^Br^G)G_2_T(TAG_3_T)_2_ in K^+^ (*antiparallel form 3*) [86]; (**e**) NMR structure of AG_3_(T_2_AG_3_)_3_ in Na^+^ (*antiparallel*) [89]; (**f**) NMR structure of (T_2_AG_3_)_3_TTA(^Br^G)G_2_T_2_A in Na^+^ (*antiparallel*) [90]; (**g**) NMR structure of T_2_AG_3_T in K^+^ (*parallel tetramer*) [92]; (**h**) NMR structure of UAG_3_T(^Br^U)AG_3_T in K^+^ (*antiparallel dimer*) [93]; (**i**) crystal structure of (TAG_3_T)_2_ in K^+^ (*parallel dimer*) [84]; the same topology was seen in equilibrium with (**h**) by NMR with TAG_3_UTAG_3_T in K^+^ [93]. Guanines: purple spheres; thymines: yellow spheres; adenines: green spheres. *Syn* guanines shown in dark purple, *anti* guanines in light purple.

If human telomeric overhangs do form into G-quadruplexes in vivo, it is also not yet known which conformation(s) of G-quadruplex are present. There has been an enormous amount of experimental effort devoted to determining the structure of G-quadruplexes formed from 4-repeat human telomeric oligonucleotides (i.e., 21–24 nt) in vitro, which has revealed the highly polymorphic nature of the structures formed by this sequence (Figure 3, reviewed in [83]). The solved intramolecular structures so far include a parallel-stranded “propeller-loop” form, an antiparallel conformation and two different “hybrid” structures in the presence of K^+^ [84,85,86,87,88], and two different antiparallel conformations and a hybrid form in Na^+^ [89,90,91]. Oligonucleotides with fewer than four telomere repeats can also form into intermolecular structures, either dimeric or tetrameric, under the right conditions [39,84,92,93]. Intermolecular G-quadruplexes at telomere overhangs could play a biological role in situations where there are associations between telomeres, such as the sister chromatid cohesion that is established at the time of DNA replication [94], or the clustering of telomeres into a “bouquet” formation that occurs in meiosis [95]. There is no experimental evidence for these possibilities as yet, although the potential for G-quadruplexes to be involved in chromosome pairing in meiosis was proposed more than 30 years ago [18].

With regards to intramolecular human telomeric G-quadruplexes, most focus has been directed towards the structures that form in K^+^, since this is considered to be the more physiologically-relevant cation [96]. The sequence at the 5′ and 3′ ends of the oligonucleotide influences which of these structures predominates in solution [87,97], but for any particular sequence, different structures coexist and interconvert at equilibrium. Single-molecule techniques such as single-molecule fluorescence resonance energy transfer (smFRET) or fluorescence-force microscopy are particularly useful for distinguishing different conformations in a mixture [98,99]; for example, the latter technique detected six different forms of the human telomeric G-quadruplex coexisting in K^+^ solution [100]. smFRET is also useful for characterizing G-quadruplex folding and unfolding kinetics, and has revealed that there are multiple folding intermediates in both K^+^ and Na^+^ solutions [99,101,102,103]; the lifetimes of some of these intermediates are sufficient that they could influence the overall population of conformations in vivo, particularly if they form preferred substrates for particular proteins.

The length of human telomeric overhangs ranges from 20–400 nt, and averages ~30 nt on telomeres replicated by leading strand synthesis and ~100 nt on lagging strand telomeres [73,74]. Many overhangs, although not all, would therefore be long enough to fold into multiple G-quadruplexes. Recent effort has therefore moved from characterization of 21–24 nt oligonucleotides, to longer sequences more reflective of the in vivo situation. Molecular dynamics simulations combined with sedimentation velocity and fluorescence measurements supported the conclusion that oligonucleotides consisting of 8 or 12 telomeric repeats form primarily into 2 or 3 consecutive hybrid-type G-quadruplexes, respectively, with no gaps between them [104,105]. On the other hand, a single-molecule force ramping assay using oligonucleotides ranging from 4 to 12 repeats supported an alternative scenario, in which G-quadruplexes randomly form along the sequence and may have single-stranded telomere repeats between them [106]. Visualization of the behavior of very long single-stranded telomeric DNA (100 nt–20 kb) by electron microscopy supports the existence of single-stranded regions between G-quadruplexes [82]. Furthermore, the DNA folded into “beads”, consisting of higher-order assemblies of G-quadruplex structures, possibly involving G-quartet formation between telomere repeats interspersed among the G-quadruplexes (Figure 2, bottom right). Each “bead” spans either 20 or 40 telomeric repeats, containing four or eight G-quadruplexes respectively, resulting in an overall 12-fold compaction of the DNA [82]. This intriguing observation suggests the capability of G-quadruplexes to effectively shield single-stranded overhangs from DNA repair and recombination activities, but it remains to be seen whether the behavior reflects that of telomere overhangs in vivo, particularly since the majority of overhangs are a lot shorter than the strands used in this study.

Other recent studies examining the structure of human telomeric G-quadruplexes have focused on efforts to reproduce physiological conditions in vitro, in particular the “molecular crowding” conditions inside a cell. Several studies found that the addition of cosolutes such as polyethylene glycol (PEG), ethanol, dimethyl sulfoxide and acetonitrile induced a transition from a hybrid-type conformation to a parallel G-quadruplex, both with short oligonucleotides and with those containing up to 20 telomeric repeats [97,107,108,109,110]. Subsequent studies, however, failed to observe a similar transition in the presence of Ficoll, 30% BSA or *Xenopus* (frog) oocyte extracts, and ascribed the structural change induced by PEG to effects of dehydration and/or direct binding of PEG to the G-quadruplex [111,112,113,114,115]. Comparison of NMR spectra of 4-repeat or 8-repeat human telomeric oligonucleotides in *Xenopus* oocyte extracts with those of known structures led to the conclusion that the physiologically-relevant forms are antiparallel and one of the hybrid forms [113]. This conclusion was supported by in-cell NMR using oligonucleotides either microinjected into *Xenopus* oocytes or introduced into human cells with a membrane-perforating enzyme [113,116]. However, these studies did not rule out the existence of a minor population of another conformation, and indeed similar in-cell NMR experiments in *Xenopus* oocytes have reported a mixture of hybrid and parallel conformations [117], as have in vitro unfolding assays using laser tweezers in the presence of BSA [118]. Furthermore, different cellular compartments have different levels of molecular crowding and cosolute compositions; the *Xenopus* extracts used for the above experiments are generally cytoplasmic rather than nuclear, and the oligonucleotide introduced into human cells was mostly located in the cytoplasm [116]. Indeed, it has been demonstrated using FRET that G-quadruplex-forming oligonucleotides introduced into human cells have different conformations in the nucleus, cytoplasm and nucleolus [119]. Also, even in the absence of crowding or dehydrating agents, very high concentrations of DNA induce a conversion of hybrid telomeric G-quadruplexes to a parallel form that is likely intermolecular [97,120,121], and divalent cations such as Ca^2+^ and Sr^2+^ can also promote formation of parallel G-quadruplexes [39,122,123]. An antibody that specifically recognizes parallel G-quadruplexes detected human telomeres both by immunofluorescence and chromatin immunoprecipitation, supporting the existence of parallel G-quadruplexes at human telomeres [124,125], although it should be remembered that these techniques do not distinguish between structures at the telomeric overhang and those forming in the normally double-stranded region of telomeres. Altogether, it can be concluded that the existence and conformation of G-quadruplexes at human telomeric overhangs in vivo remains an open question.

## 4. Do G-Quadruplexes at Overhangs Have a Telomere-Capping Function?

The almost-universal conservation of a G-rich sequence at telomeres has long promoted speculation that there is a selective evolutionary advantage for the formation of G-quadruplexes at telomeres. It has been postulated that G-quadruplexes may have played a role in the evolution of linear chromosomes [126,127]; it is possible that breakage of a circular chromosome was repaired by acquisition of non-LTR-retrotransposons, which has been shown to occur in yeast or mammalian cells if other DNA repair pathways are defective [128,129,130]. Multiple such events could result in tandemly repeated sequences, and once such repeats are able to form secondary structures, these structures could form a protective cap on the chromosome end, rendering repair unnecessary [127]. The earliest telomeres are likely to have been maintained by recombination between repeats, possible involving rolling-circle amplification of a t-loop structure [77,131]. Telomerase evolved early in eukaryotic evolution, most likely from a reverse transcriptase encoded by non-LTR-retrotransposons [132,133]. In a fascinating evolutionary twist, some arthropods, such as *B. mori* and *Tribolium castaneum*, have low levels of telomerase and have regained insertion of non-LTR-retrotransposons among their telomerase-transcribed repeats, but in a manner that preserves the G/C strand bias [134,135]. *Drosophila* species have dispensed with telomerase altogether, instead relying on transposition of tandem repeats of retrotransposons for telomere maintenance [136,137]. There is some evidence that parts of these retrotransposons can form G-quadruplex structures [138], bolstering the idea that G-quadruplex formation is a conserved property of telomeres.

There are some species, however, whose telomere repeat sequences do not form stable G-quadruplexes. The two-guanine repeats of *B. mori* and the nematode *Ascaris lumbricoides* (GGTTA and GGCTTA, respectively) fold into G-quadruplexes that are in equilibrium with hairpin-duplexes [24], and those of *C. elegans* appear to only form hairpins [139,140]. This has led to the proposition that is the ability of G-rich telomere repeats to form stable secondary structures that confers an evolutionary advantage, regardless of whether those structures are G-quadruplexes or something else [139]. If true, this supports the notion that G-quadruplexes or G-rich hairpin structures play a positive role at telomeres, providing the protective function that is central to telomere identity.

**Figure 4:**
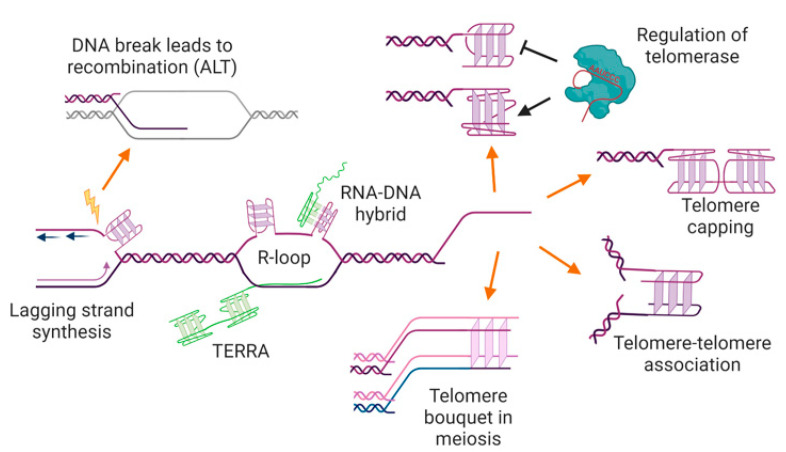
Potential locations, functions, and consequences of G-quadruplexes at telomeres. G-quadruplexes may form in the single-stranded telomere overhang, where they may positively or negatively regulate telomerase and/or have a capping function, preventing access to the DNA repair machinery. G-quadruplexes at overhangs may also mediate interactions between two telomeres (e.g., during sister chromatid cohesion, or in the macronucleus of ciliated protozoa), or be involved in telomere clustering in meiosis. G-quadruplexes could also form in the double-stranded region of the telomere during DNA replication or transcription, where they may trigger genome instability and/or recombination-mediated telomere maintenance. The RNA transcribed from telomeres, TERRA, can also form into G-quadruplexes, either unimolecular or as an RNA-DNA hybrid. See text for details and references. Figure created with BioRender.com.

To date, there is only a small amount of experimental evidence for a protective “capping” function for G-quadruplexes at telomeres (Figure 4). One key study used *S. cerevisiae* mutants with a defect in the telomere capping protein Cdc13, and found that multiple different treatments predicted to result in an increase in G-quadruplex formation (stabilizing ligands, expression of G4-stabilizing proteins, deletion of the gene encoding a G4-resolving protein) resulted in the rescue of the growth defect of the mutant [141]. This provides evidence that G-quadruplexes can substitute for the protective function of Cdc13. More recently, it has been demonstrated that Cdc13 mutant yeast have an increase in the amount of G-quadruplexes at telomeres, measured using ChIP with the BG4 antibody [26], providing evidence that even in the absence of G4-stabilizing treatments, G-quadruplexes can step in to take the place of Cdc13 at telomeres.

## 5. G-Quadruplexes at Telomeric Overhangs: Effects on Their Elongation by Telomerase or ALT

**Figure 5:**
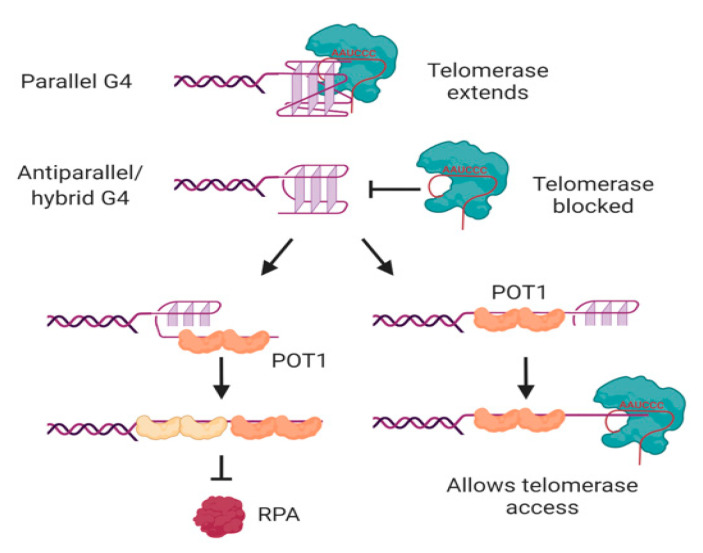
Interactions of telomerase, POT1 and RPA with human telomeric G-quadruplexes. Telomerase can bind and extend parallel, but not antiparallel or hybrid, G-quadruplexes. POT1 binds to antiparallel or hybrid G-quadruplexes through a mechanism in which G4 unfolding precedes “trapping” of the unfolded DNA by POT1. The two OB folds of each POT1 molecule bind to consensus binding site TTAGGGTTAG; sequential binding of two POT1 molecules therefore coats the 4-repeat telomeric DNA (left). Although RPA also has the ability to unwind G-quadruplexes, POT1 competes with this activity. If binding of POT1 occurs at the 5′ region of the DNA, the 3′ tail can form a substrate for telomerase (right). Not shown is POT1’s binding partner TPP1, which also influences G4 unwinding dynamics and telomerase activity. See text for details and references. Figure created with BioRender.com.

In human cells, the major protective single-stranded binding protein at telomeric overhangs is POT1 [142]. The potential formation of G-quadruplexes at overhangs would therefore be in competition with POT1 binding; indeed, POT1 has been shown to unwind intramolecular G-quadruplexes in order to bind to telomeric DNA [143,144]. POT1 unwinds G-quadruplexes through a conformational selection mechanism in which G-quadruplex unwinding occurs prior to POT1 binding [145]; each of the OB folds of two POT1 molecules then binds to one telomeric repeat of the 4-repeat G-quadruplex in a stepwise manner (Figure 5) [146,147]. Another G4-unwinding protein that is known to localize to telomeres is replication protein A (RPA) [148], which plays a major role in activating the DNA damage response to single-stranded DNA [149]. If human telomeric oligonucleotides are folded into G-quadruplexes, the ability of POT1 to trap them as they unfold out-competes the ability of RPA to unfold the G-quadruplex [150], illustrating the protective function of POT1 at telomeres.

POT1-mediated G-quadruplex unfolding also affects the ability of telomeric sequences to be extended by telomerase, at least in vitro [143]. It was first demonstrated using telomerase from three different species of ciliated protozoa that oligonucleotides folded into antiparallel intramolecular G-quadruplexes [151,152,153] do not form good substrates for telomerase [154,155]. This is also the case for human telomerase; the antiparallel and hybrid G-quadruplexes formed by a 4-repeat oligonucleotide in K^+^ solution [85,86,87,88] are poor substrates for telomerase [39,143,156]. Binding of telomerase to the DNA is inhibited by these conformations of G-quadruplex [157,158], and the attempted extension of the DNA by telomerase is non-processive [39,143]. POT1 can restore the ability of telomerase to access these substrates by unfolding the G-quadruplex and trapping the DNA in a linear form with a protruding tail (Figure 5) [143]. The dynamic folding of G-quadruplexes and their unfolding by POT1 may therefore act as a regulatory mechanism, controlling access of telomerase to the telomere.

However, not all G-quadruplexes are refractory to telomerase extension. Parallel G-quadruplexes, whether intermolecular or intramolecular, can be bound and extended by both ciliate and human telomerase [39,155,159,160]. The 3′ end of the DNA aligns correctly with the RNA template of telomerase [39,159], and telomerase then unwinds the rest of the G-quadruplex as it extends the DNA [160]. It should be noted, however, that the preferred substrate of telomerase is linear DNA; the affinity for parallel G-quadruplexes is about 5-fold lower than their corresponding linear oligonucleotides, and accommodation of the relatively bulky G-quadruplex in the active site results in a decrease in the affinity of telomerase for incoming nucleotides [39,159]. Nevertheless, the ability of telomerase to bind, unwind and extend parallel G-quadruplexes implies that it is able to deal with these structures if it encounters them in vivo. Whether this is most likely to be in intramolecular form at a telomeric overhang, or in intermolecular form through the association of telomeres with their sister chromatids or in a meiotic bouquet, remains to be determined.

G-quadruplexes also play a functional role in processive elongation of DNA by telomerase. Telomerase activity can be measured in vitro with a direct primer extension assay, in which telomerase extends a telomeric primer in the presence of radiolabeled nucleotides, and the products are separated by high-resolution gel electrophoresis and visualized by phosphorimaging. This enables assessment of “processivity”, i.e., the ability of telomerase to repetitively add repeats to a single substrate molecule. It was noted early on that the presence of K^+^ in such activity assays resulted in an increase in products that correspond to multiples of four telomeric repeats; this suggests that G-quadruplex formation in the extended DNA products causes dissociation of the product from the enzyme [161]. This has been reinforced by the inclusion of G4-stabilizing ligands in telomerase primer extension assays; many (but not all) of these compounds cause a similar periodicity, in which products with enough repeats to form G-quadruplexes are overrepresented on the gel [156,162,163,164]. The activity of ciliate telomerase was examined in the presence of nucleotide analogues that can be incorporated into the telomerase product DNA, but would impair secondary structure formation; these analogues completely inhibited the ability of telomerase to extend the product beyond four repeats, demonstrating that formation of a secondary structure in the product DNA is necessary for the process of telomerase translocation [165]. This was confirmed by recent kinetic and smFRET studies that demonstrated that G-quadruplex formation in telomerase product DNA enhances the rate of product dissociation from telomerase, but also the rate of translocation, resulting in an overall increase in the length of products in conditions that support G-quadruplex formation [166]. An assay that measures the dynamics of telomerase product extension and release using optical tweezers confirmed the positive effect of G-quadruplex formation in the product DNA on overall extension rate, and showed that the formation of these G-quadruplexes is dynamic [167], which explains why overstabilization of these structures with small molecules reduces the processivity of telomerase.

The initial impetus for the development of G4-stabilizing ligands as potential cancer therapeutics was the prospect that such molecules would inhibit telomerase, which is necessary for the unlimited division of cancer cells. However, the focus of the field has moved away from this aim in recent years, for several reasons. Firstly, while many G4-stabilizing ligands do have the ability to interfere with telomerase processivity, as well as interfering with telomerase binding and extension of 4-repeat oligonucleotides that form into antiparallel or hybrid G-quadruplexes [156], this property has not been conclusively demonstrated for all ligands. Many studies have used a PCR-based activity assay (known as the TRAP assay) for assessing ligand effects on telomerase, which is an inappropriate assay for this purpose, since the PCR step may also be inhibited by the ligand [156]. Even those studies using direct primer extension assays usually use a 3-repeat telomeric primer, which does not assay the ability of telomerase to use a substrate folded into a stable intramolecular G-quadruplex. Secondly, any effects of ligands on telomerase extension of telomeres in vivo is likely to be far outweighed by the direct effects of G-quadruplex stabilization on telomere capping, as discussed above, and effects on G-quadruplexes throughout the genome. This may not necessarily be a bad thing, since telomere uncapping or genomic DNA damage may cause more rapid effects on cancer cell viability than the delayed senescence caused by gradual telomere shortening. Thirdly, human telomerase does have the ability to extend some G-quadruplexes [39], and its ability to do so is not always inhibited by G4-stabilizing ligands [160]. Therefore, recent attention has focused on nontelomeric mechanisms by which G4-stabilizing molecules may cause death or growth arrest of cancer cells [168,169,170]. The relative contribution of telomeric vs. nontelomeric effects on reduced cancer cell viability remains to be determined for any particular G4-stabilizing ligand.

Not all human cancers use telomerase to elongate their telomeres; some have activated a recombination-based mechanism (known as ALT for Alternative Lengthening of Telomeres), that also provides a back-up pathway for telomere elongation in telomerase-negative yeast mutants [171,172,173,174]. There is some evidence that treatment of human cells using ALT with G4-stabilizing ligands increases the extrachromosomal DNA that is produced as part of the ALT mechanism [175,176], and also increases ALT-mediated telomere synthesis ([177], and Bryan et al., unpublished data). This may be a result of DNA breaks caused by ligand-induced replication fork stalling, that can trigger recombination-mediated DNA synthesis in repetitive DNA (Figure 4) [178,179]. If recombination-mediated DNA synthesis represents the ancestral mechanism for maintenance of the ends of linear chromosomes [77], it is tempting to speculate that the evolutionary selection for G-rich tandem repeats arose due to the stimulation of telomeric recombination by secondary structures such as G-quadruplexes.

## 6. G-Quadruplexes in the Double-Stranded Portion of Telomeres

Most discussion of the presence of G-quadruplexes at telomeres focusses on their potential existence in the 3′ overhang, since this part of the telomere is presumed to be single-stranded, at least transiently. However, it needs to be remembered that the double-stranded region of the telomere is much larger than the overhang, by up to 100-fold in human cells, and there is frequent separation of the two strands during the processes of DNA replication and transcription. Immunofluorescence with the G-quadruplex antibody BG4 showed a greater number of foci throughout the genome during S phase, and these foci were reduced by treatment with the DNA polymerase inhibitor aphidicolin, demonstrating that G-quadruplexes form during DNA replication in human cells [38]. ChIP-sequencing experiments with the BG4 antibody, in unperturbed human cells, found a significant localization of G-quadruplexes in transcriptionally active euchromatin [61], indicating the likely formation of G-quadruplexes in the non-transcribed strand during transcription [180]. The proportion of G-quadruplexes detected at telomeres that are localized at sites of replication, sites of transcription, or single-stranded overhangs remains to be determined.

Telomeres are particularly problematic areas of the genome for the DNA replication machinery [181,182,183,184,185]; this may be at least partly due to their propensity for secondary structure formation. During normal DNA replication, single-stranded regions are transiently exposed on the lagging strand between the replisome and the replicative helicase [186,187], providing an opportunity for spontaneous formation of G-quadruplexes (Figure 4). Since telomeres are mostly replicated unidirectionally from origins in the subtelomeric region [188], the G-rich strand of the telomere is usually the template for lagging-strand synthesis. Treatment of cells with G4-stabilizing ligands has demonstrated the potential for G-quadruplexes to impede DNA replication (reviewed in [189]). Several different ligands, including telomestatin, pyridostatin, and the bisquinolium compounds 360A and PhenDC3, result in stalling of replication forks in telomeres of human and mouse cells, resulting in a distinctive disrupted pattern of binding of telomere probes, known as a “fragile telomere” phenotype [49,50,169,190]. It remains to be determined to what extent G-quadruplexes form at replication forks in untreated cells, but if they do occur, they are likely to be an impediment to progression of the fork.

However, analysis of the speed of replication fork progression using single molecule combing assays of DNA from human cells found no difference in rate between telomeric and nontelomeric regions [188]. Also, analysis of the effect of G-quadruplexes on telomeric DNA replication in human cells using a shuttle vector mutagenesis assay showed a greater level of disruption when the G-rich strand was the leading rather than the lagging strand, contrary to the expected greater opportunity for G-quadruplexes to arise during lagging strand replication [191]. These studies illustrate that human cells are endowed with robust mechanisms to deal with G-quadruplexes that arise during replication. Indeed, many studies have found that depletion of helicases known to unwind G-quadruplexes, such as BLM, WRN, FANCJ and RTel1, results in a “fragile telomere” phenotype, telomeric lagging strand defects, or slowed replication through telomeres in combing assays (reviewed in [189,192]), demonstrating that part of the functions of these proteins is to keep replication moving through telomeres or other G-rich regions. Importantly, a greater number of G-quadruplexes were detected by immunostaining with the BG4 antibody in the absence of BLM, WRN or RTel1, particularly at telomeres in the case of BLM and WRN, demonstrating that G-quadruplex unwinding is involved in the role of these proteins in facilitating telomere replication [193,194]. An increase in the number of G-quadruplex foci, both at telomeres and across the genome, was also observed upon depletion of the human CST (CTC1–STN1–TEN1) complex, an RPA-like complex that binds single-stranded DNA and facilitates DNA replication [125]. The nucleases DNA2 and EXO1 are also involved in removing G-quadruplexes that impede replication of telomeres [190,195].

G-quadruplexes can also form where double-stranded DNA has been resolved to allow transcription to occur, a region known as an R-loop (Figure 4) [61,180]. Conversely, stabilization of G-quadruplexes can increase the formation of R-loops at the same location [196,197]. Despite showing characteristics of heterochromatin, telomeres of humans and many other organisms are transcribed into telomeric repeat-containing RNA (TERRA) [198,199]. TERRA is always transcribed from the C-rich strand, theoretically leaving the G-rich strand free to form secondary structures. It has recently been shown that depletion of TERRA with antisense oligonucleotides causes a decrease in G-quadruplex foci detected at telomeres with the BG4 antibody in G1-synchronized human cells, providing the first direct evidence that G-quadruplexes can form at telomeric R-loops [125].

Furthermore, TERRA consists of G-rich telomere repeats, so can itself form into stable parallel G-quadruplexes [200,201,202,203]. Since TERRA molecules remain localized to the vicinity of telomeres [198], it is possible that some of the G-quadruplex signals at telomeres are due to TERRA rather than telomeric DNA. Introduction of TERRA molecules tagged with fluorescent reporter dyes into human cells showed that TERRA G-quadruplexes can form in cells, and are localized to telomeres [204]. The TERRA may be tethered to telomeres via interactions with telomere proteins such as TRF2 [205], or it may form an RNA-DNA hybrid G-quadruplex (Figure 4) [206,207]. The biological implications of G-quadruplex formation in TERRA remain to be fully elucidated; one recently-described function is TERRA-mediated recruitment of the heterochromatin-associated protein HP1α, which binds specifically to parallel G-quadruplexes [208].

## 7. Conclusions

In recent years, the development of tools such as G-quadruplex-specific antibodies and technologies such as in-cell NMR are providing exciting confirmation that telomeric DNA does indeed have the capacity to form into G-quadruplexes and other secondary structures in the cells of humans and other organisms; however, many questions remain. The proportion of the observed G-quadruplexes that are present at telomeric 3′ overhangs, compared to those forming in the normally double-stranded region of telomeres, is unknown. The conformation of human telomeric G-quadruplexes, whether at the overhangs or internally, is the subject of heated debate. There is evidence that telomeric G-quadruplexes can perform a protective capping function in yeast, and are involved in telomere-telomere associations in ciliates, but these concepts have not yet been examined in other organisms. Telomerase can extend parallel G-quadruplexes but not other conformations, but the in vivo circumstances in which it encounters the former structures have not yet been elucidated. And finally, the degree to which individual G4-stabilizing ligands cause arrest or death of cancer cells through a telomere-related mechanism, compared to effects elsewhere in the genome such as at gene promoters, remains to be determined. For both fundamental biological understanding of genome maintenance, and potential translational applications of this knowledge, these questions remain exciting areas for future study.
